# Assessing Health Data Security Risks in Global Health Partnerships: Development of a Conceptual Framework

**DOI:** 10.2196/25833

**Published:** 2021-12-08

**Authors:** Juan Espinoza, Abu Taher Sikder, James Dickhoner, Thomas Lee

**Affiliations:** 1 Department of Pediatrics Children's Hospital Los Angeles Los Angeles, CA United States; 2 Keck School of Medicine University of Southern California Los Angeles, CA United States; 3 Innovation Studio Children's Hospital Los Angeles Los Angeles, CA United States; 4 Department of Surgery Children's Hospital Los Angeles Los Angeles, CA United States

**Keywords:** health information technology, low- and middle-income countries, low income, conceptual framework analysis, framework method, data security, decision-making, database, information use, misuse, global health, security

## Abstract

**Background:**

Health care databases contain a wealth of information that can be used to develop programs and mature health care systems. There is concern that the sensitive nature of health data (eg, ethnicity, reproductive health, sexually transmitted infections, and lifestyle information) can have significant impact on individuals if misused, particularly among vulnerable and marginalized populations. As academic institutions, nongovernmental organizations, and international agencies begin to collaborate with low- and middle-income countries to develop and deploy health information technology (HIT), it is important to understand the technical and practical security implications of these initiatives.

**Objective:**

Our aim is to develop a conceptual framework for risk stratification of global health data partnerships and HIT projects. In addition to identifying key conceptual domains, we map each domain to a variety of publicly available indices that could be used to inform a quantitative model.

**Methods:**

We conducted an overview of the literature to identify relevant publications, position statements, white papers, and reports. The research team reviewed all sources and used the framework method and conceptual framework analysis to name and categorize key concepts, integrate them into domains, and synthesize them into an overarching conceptual framework. Once key domains were identified, public international data sources were searched for relevant structured indices to generate quantitative counterparts.

**Results:**

We identified 5 key domains to inform our conceptual framework: State of HIT, Economics of Health Care, Demographics and Equity, Societal Freedom and Safety, and Partnership and Trust. Each of these domains was mapped to a number of structured indices.

**Conclusions:**

There is a complex relationship among the legal, economic, and social domains of health care, which affects the state of HIT in low- and middle-income countries and associated data security risks. The strength of partnership and trust among collaborating organizations is an important moderating factor. Additional work is needed to formalize the assessment of partnership and trust and to develop a quantitative model of the conceptual framework that can help support organizational decision-making.

## Introduction

### Background

Health information technology (HIT) refers to electronic health records (EHRs), patient portals and other software platforms, public health databases, hardware devices, and technology systems, which contain a wealth of information used for patient care and resource allocation. According to a 2016 World Health Organization (WHO) global survey on eHealth, the adoption of EHR systems had increased by 46% in the previous 5 years [1]. Increasing numbers of low- and middle-income countries (LMICs) are implementing HIT as they continue developing their health care infrastructure [2,3]. In many cases, these systems are being implemented in collaboration with foreign academic institutions, health care systems, and nonprofit or research organizations [4,5]. However, there are unique organizational, technical, functional, educational, and ethical challenges that require meticulous consideration—especially with respect to their security implications [6].

In many LMICs, legal, regulatory, and technical frameworks around HIT are undeveloped [6]. Furthermore, the sensitive nature of health data (eg, ethnicity, reproductive health, sexually transmitted infections, and lifestyle information) can have significant impact on individuals if misused [7]. The combination of developing legal frameworks and decreased ability of public institutions to protect individuals may create a particularly vulnerable environment for HIT and health data. A framework to understand and stratify the risk associated with HIT may be beneficial to organizations engaging in global health partnerships that generate significant amounts of health data.

**Figure 1:**
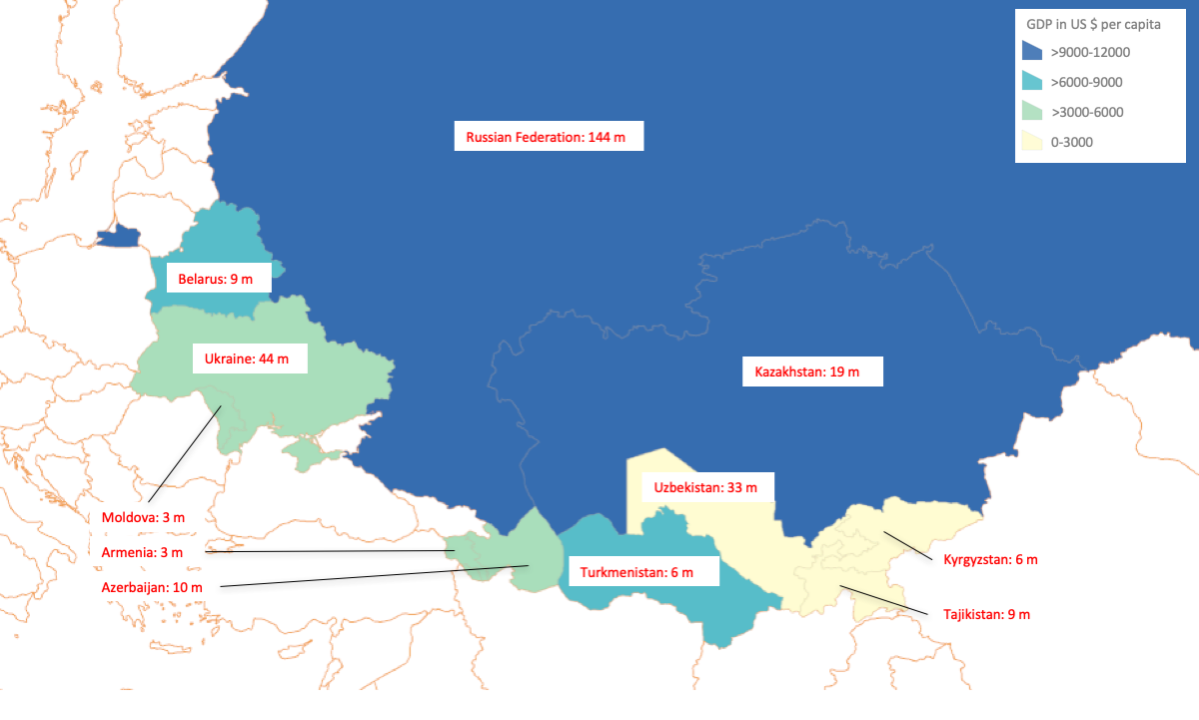
A map of Commonwealth of Independent States countries located in Eastern Europe and Central Asia. The color gradient shows 2019 gross domestic product per capita in US dollars. The population of the country (in millions) is noted next to the name. GDP: gross domestic product.

Our research team has been focused on international clinical and research partnerships in Armenia. As we have engaged in the process of designing and deploying a safe, scalable health data platform in Armenia, other countries in the region have expressed interest in implementing similar systems. This geopolitical region is home to the Commonwealth of Independent States (CIS), an intergovernmental organization of 11 post-Soviet countries: Armenia, Azerbaijan, Belarus, Kazakhstan, Kyrgyzstan, Moldova, Russia, Tajikistan, Turkmenistan, Ukraine, and Uzbekistan (Figure 1) [8]. These countries inherited the Soviet Union’s Semashko health system and many of its flaws in transitioning to a modern primary care model, including premature mortality, variable quality of care, poor noncommunicable disease management, and high out-of-pocket payments [9-11]. Rising social and economic challenges such as inequality and the cost of funding public health are also significant concerns [9]. To address these concerns, CIS member states have embarked on health care reform efforts to improve their health care systems, including deploying HIT [9,12].

### Need for a Framework

As our organization considers new partnerships among other CIS members, the need for a framework to understand potential HIT security risks has become more pressing. Given the lack of an existing risk stratification framework to assess and consider the security vulnerabilities with implementing HIT in LMICs, we decided to create our own. In this paper we present a conceptual approach to developing such a framework and propose a variety of indices that could be leveraged to inform each subdomain. Finally, we outline our proposed next steps to seek consensus and finalize quantitative and qualitative versions of the framework.

## Methods

### Literature Review

We reviewed the literature to identify relevant publications by searching PubMed, Ovid MEDLINE, Google Scholar, and Google using the search terms presented in Textbox 1. All articles were identified as relevant by at least 2 authors (JE, ATS, or JD). References from relevant articles were also reviewed. Original research, reviews, editorials and commentaries, position statements, white papers, and industry and nongovernmental organization reports were included. Finally, the websites of international agencies such as the WHO, the United Nations, and the World Bank were reviewed for relevant data sources and publications.

**Literature search terms by concept type.**

**Search terms**

- Geographic terms
  - Armenia
  - Europe
  - Asia
  - Soviet
  - Commonwealth of Independent States (CIS)
  - Low- and middle-income countries (LMIC)
  - Developing countries
  - Global health
  - International
- Technology terms
  - Data
  - Health data
  - Patient data
  - Information
  - Health information
  - Patient information
  - Electronic health records (EHR)
  - Electronic medical records (EMR)
  - Health information technology (HIT)
  - Personal health records
  - Database
- Theme terms
  - Security
  - Risk
  - Partnership
  - Breach
  - Trust
  - Safety
  - Health system
  - Health policy
  - Legal

### Conceptual Framework Development

**Figure 2:**
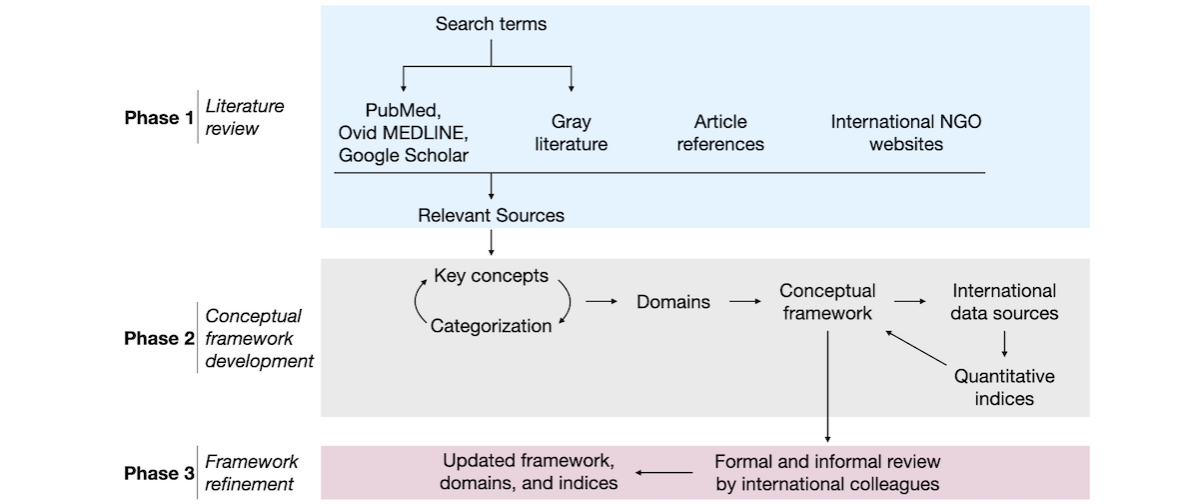
A visual representation of our approach to developing our conceptual framework, from literature review to refinement. NGO: nongovernmental organization.

A conceptual framework is a “network of...interlinked concepts that allow for a comprehensive understanding of a phenomenon or phenomena [13].” Our conceptual framework development process borrowed iterative techniques from both the framework method [14] and conceptual framework analysis [13]. First, all literature sources were extensively reviewed for key concepts. By categorizing and recategorizing these key concepts iteratively as more literature was reviewed, the team conceptualized them into domains that were then synthesized to develop an overarching conceptual framework using the coding methodology outlined by Gale et al [14]. Once key domains were identified, public international data sources were searched for relevant structured indices to generate a quantitative counterpart (Figure 2).

### Expert Input and Refinement

Our conceptual framework, key domains, and indices were shared formally and informally through email, conversations, and presentations with international colleagues in health care, information technology, informatics, health policy, and international relations. Feedback was collected and an iterative approach was used to refine the model and the indices included.

## Results

### Key Domains and Indices

Our analysis resulted in an inventory of concepts that aggregated into 5 key domains that make up our conceptual framework for assessing health data security considerations in global health partnerships:

1. State of HIT
2. Economics of Health Care
3. Demographics and Equity
4. Societal Freedom and Safety
5. Partnership and Trust

For each of the first 4 domains, we identified publicly available indices that could be used to provide qualitative or quantitative insights. For the fifth domain, *Partnership and Trust*, we created a self-assessment tool that would allow an organization’s members to take into consideration the specifics of their project, their experience on the ground, and their in-country partners.

### State of HIT

#### Overview

HIT refers to all the electronic systems used by care providers, public health workers, patients, researchers, and others to manage health information [15]. It includes EHRs, other software platforms, hardware devices, and technology systems [16]. HIT systems are a critical component of several aspects of health care, including care delivery, billing, medicolegal liability, research, and health policy [17,18]. HIT use is influenced by factors such as cost, technical feasibility, and regulation [3].

The *State of HIT* domain evaluates a health system’s maturity in terms of its logistic, technological, and regulatory progress within a country. The legal structure and regulatory framework around HIT and medical ethics directly influence the viability and progress of HIT [19]. As suggested by Luna et al [19], overcoming legal and ethical challenges, interoperability issues, and technical security vulnerabilities greatly affects the implementation of HIT.

#### Rationale for Indices

Multimedia Appendix 1 [6] presents the proposed indices of this domain categorized into 3 broad subdomains:

1. EHR deployment considers the presence of a national EHR system’s existence, regulation, and location. National EHR systems require a high level of commitment as well as technical and financial resources [3,20]. This implies a certain level of investment, commitment, and organizational sophistication and provides insight into the groundwork laid by the country that affects the long-term viability and integrity of HIT and health data [20,21]. Were at al [20] have commented on the unsustainability of expensive implementations and the need for sustainability to be built into EHR implementations, underscoring the demand that EHRs place on technical and financial resources. This subdomain also considers EHR implementation location (in primary, secondary, or tertiary facilities), which gives an understanding of the level of penetration of the technology in the market. Overall, EHRs are often the first, most expansive, and most expensive component of HIT; as such, they serve as reasonable proxies for HIT overall, including technical and logistical risks with implementing new technologies, where other measures are not available. The indices in Multimedia Appendix 1 [6] were gathered from the Electronic Health Record section of the Atlas of eHealth Country Profiles compiled by the WHO as “Yes, No, Not applicable [6].”
2. eHealth Foundations refers to the national strategy, policies, and funding information for eHealth. These factors are critical to determining the success or failure of HIT and can provide valuable insights into risk stratification of a given environment [22]. These indices were gathered from then eHealth Foundations section of the Atlas of eHealth Country Profiles compiled by the WHO as “Yes, No, Not applicable [6].”
3. Legal Frameworks for eHealth refers to the policies, regulation, and jurisdiction governing the use, quality, sharing, and ownership of health data. Medicolegal concerns are universal, complex challenges of HIT that affect use and the experience of patients, providers, and other stakeholders [20,23]. The existence of legal solutions or mechanisms to address these concerns can provide insight into the preparedness of a country to mitigate HIT and data breaches as well as suggest the recourse that individuals have should a breach occur. For example, Palabindala et al [23] mention that Health Insurance Portability and Accountability Act compliance requires substantial legal, technical, and logistical efforts that ensure the establishment of appropriate measures for unfortunate events. These indices were gathered from the Legal Frameworks for eHealth section of the Atlas of eHealth Country Profiles compiled by the WHO as “Yes, No, Not applicable [6].”

### Economics of Health Care

#### Overview

The *Economics of Health Care* domain aims to quantify a country’s overall investment in health care. This domain evaluates a country’s financial and resource investment in health care access, delivery, services, and technology. Government investment directly affects infrastructure, quality of care, and affordability for patients and providers, which in turn significantly influences the successful and sustainable implementation of HIT, especially in LMICs [19,24,25]. Without proper allocation of funding, resources, care standards, and cost-effectiveness for stakeholders, there is a greater chance of failure in terms of long-term success and scalability [20]. As discussed by Luna et al [19], financial and technical sustainability is an important element of HIT integration and therefore needs to be addressed before implementation.

#### Rationale for Indices

This domain can be further evaluated as 4 subdomains; the individual indices have been presented in Multimedia Appendix 2 [26-43]:

1. Health Care Expenditure considers gross domestic product (GDP), contextualized GDP for health care, and health care expenditure. These indices were gathered from data by the World Bank on GDP, GDP per capita, GDP growth percentage, health care spending percentage per GDP, and health care spending per capita in US dollars, US dollars per capita, or percentages depending on the metrics involved [26-31]. Indices such as recontextualized GDP values and health care spending per capita have been described by Naik et al [44] as macroeconomic determinants of health that ultimately influence HIT integration and management. Insufficient health care funding increases the chances of failure of HIT implementation and health data management [20]. Health expenditure and GDP spending on health care thus become potential proxies for risk assessment.
2. Health Care Structure considers both infrastructure and system indices such as hospital beds, doctors per capita, health care access, and the existence of a public health care system. These indices were gathered from data sets by the World Bank on doctors per capita and hospital beds, the Global Burden of Disease (GBD) index on health care access and quality, and the US Social Security Administration on private versus public health care systems as numbers (per 1000 people), indices (1-100), and binary values [32-35]. As these indices directly measure access and the availability of crucial health care resources, they provide insight into the economic and material context of health care. Lower scores for these indices may highlight a higher risk of not having adequate finances and resources for implementing and managing HIT systems [3]. Specifically, Akhlaq et al [3] identified infrastructure, finance, organization, and data management as key factors in the adoption and management of HIT.
3. Health Care Cost considers out-of-pocket fees and universal health coverage. These indices were gathered from the 2017 Global Monitoring Report by the World Bank on universal health coverage as values (0-100) measuring affordability and data sets by the World Bank on out-of-pocket costs as a percentage of total universal coverage [36,37]. These indices are important because they provide insight into the patient-level microeconomic context. Cost-effectiveness and financial viability for patients directly affect access, use, and long-term potential of health care services and resources, including HIT [19]. A lack of affordable health care can create risks for the overall success of health HIT.
4. Health Care Quality is intended to evaluate overall health care system performance through process and outcome measures such as health performance index, infant and maternal mortality, life expectancy, immunization rates, and diarrheal disease rates. These indices were gathered from data sets by GBD collaborators, the WHO, the Central Intelligence Agency, and the GBD database as an index from 1 to 191, deaths per births, age of death, and percentage immunized [38-43]. The indices listed in Multimedia Appendix 2 [26-43] are frequently used in the literature and by international organizations to measure overall health care quality [25,45,46]. Measuring health care quality provides a lens through which to interpret the economic inputs of a health care system. Major discrepancies between economic inputs and health care quality outcomes may be a cause for concern because these may result from a variety of issues, including health care administration, system stability, and inadequate data collection and reporting. All of these would be factors that may affect health data security.

### Demographics and Equity

#### Overview

The *Demographics and Equity* domain aims to describe the relevant population and possible disparities involving health care. Understanding how patients from different social, economic, ethnic, and cultural backgrounds experience HIT is important for any global health data partnership because these differences can drive care disparities [25,47]. Gathering together concepts of population demographics, social structure, and community development provides a starting point to gain necessary insights. These social and demographic variables help contextualize pragmatic concerns surrounding patient privacy, access, health discrepancies, and improper use of health data [20]. Increased digitization of health care has several advantages such as public health surveillance during COVID-19, but this same surveillance infrastructure has implications for civil liberties and governance that affect marginalized groups differently [48]. For example, the Social Science Research Council states that Black and Brown communities are subject to disproportionate police surveillance and may be unable to opt out of medical tracking and monitoring systems [48]. Being aware of these issues in the local context is an important component of responsible data stewardship.

#### Rationale for Indices

Multimedia Appendix 3 [49-61] presents the indices of this domain categorized into 3 broad subdomains:

1. Population metrics include information about the density and structure of the population. The indices were gathered from data by the World Bank on population age structure as percentages and from the United Nations on rural and urban population density in thousands [49-52]. These population metrics affect HIT in a variety of ways. For example, countries with larger populations may require more costly efforts to ameliorate data misuse [62,63]. In addition, differences in age structure and trends may affect the demand and risk of health services and data technology [64-66]. As noted by Knickman and Snell [64], the Baby Boom generation is expected to double by 2030 and will require substantially more health-related resources. The increasing financial demand and use of health data may affect the technical and logistical risks associated with HIT.
2. Social Structure includes information about wealth inequality, poverty, decentralization, and public trust. The indices were gathered from data by the World Bank, a policy paper by World Bank affiliates titled How Close Is Your Government to its People, and Edelman, a global communications firm, as either a percentage or score (1-100), as appropriate [53-56]. Wealth inequality and poverty data provide insights into the economic aspect of inequity. Decentralization has long been advocated by international development agencies to improve health system performance in LMICs [67]. A recent literature review showed limited empirical data to support this approach, but as a dominant theory in international development, it should still be considered [68]. A lack of public trust in government and health care systems can lead to poor patient compliance with public health guidance, delaying seeking care, and withholding of critical information from providers [69]. This can lead to incomplete or unreliable data. Low public trust can be a symptom of either systematic failures of health systems or breaches of trust at the interpersonal level, both of which should be taken into consideration when discussing data privacy and security.
3. Community Development includes information on human development, access to electricity and the internet, literacy rate, and social media penetration. The Human Development Index is a composite measure developed by the United Nations that quantifies the capability of an individual to live a long and healthy life and acquire resources for a basic standard of living as a value from 0 to 1 [57]. Other descriptors of community development that focus on the ability of a community to meaningfully leverage technology are also included. Data sets from the World Bank on internet subscribers, access to electricity, and literacy were gathered as a number (1-100) or percentage as appropriate [58-60]. Social media penetration was gathered from Statcounter Global Stats as a percentage [61]. Basic resources such as electricity and the internet are necessary to meaningfully interact with HIT. This is true at the level of health care facilities as well as at the individual level [2]. Settings with limited access to the internet and electricity may not be able to implement a wide variety of privacy and security tools such as two-factor authentication. Limited literacy can be a barrier to people’s ability to use and access technology and data, which may make them more vulnerable to exploitation [70,71]. This increases the human cost of inadequate privacy and security in health care. More specific concepts such as health and technology literacy may be relevant and should be explored further [70,71].

### Societal Freedom and Safety

#### Overview

Societal freedom is the liberty of an individual to function in society without coercion; the Cato Institute defines this as “the dignity of an individual [72].” As an ever-present societal factor, it influences aspects of health care both directly and indirectly [20,24,47,73]. The *Societal Freedom and Safety* domain aims to quantify the absence of coercive societal constraints on individual freedom within a country as well as the robustness of civil and political liberties; it includes concepts such as liberty of expression, social organization, and lawfulness. Overall, countries with more freedom (democratic) have more robust health care systems and lower mortality than countries with less freedom (autocratic) [74,75]. Factors such as the rule of law and the influence of civil society affect health and health outcomes [76-78]. Pinzon-Rondon et al [76] found that adherence to the rule of law is associated with a healthier population, higher life expectancy, and lower adverse health outcomes. These social parameters provide insights into how likely a malicious data breach might be; whether the threat to personal sensitive data might come from government or nongovernment actors; and how vulnerable individuals may be to adverse social, financial, and legal consequences in case of a breach of privacy with respect to their personal health data.

#### Rationale for Indices

Multimedia Appendix 4 [72,79] presents the indices of this domain along with an overall rank and score:

1. Personal Freedom includes information on the rule of law, safety and security, religious freedom, assembly and association, expression, and identity and relationship. These indices were gathered from the Human Freedom Index 2019 by the Cato Institute as an index value between 0 and 1 [72]. Personal freedom is important to consider for data security because it highlights societal challenges with data. For example, religious hostility and persecution, surveillance of expression and information, geopolitical concerns, and stigma toward historically marginalized groups may increase the risk associated with access to identifiable health information [7,80]. In a 2010 report by the London School of Economics, the authors stressed the importance of social context and appropriate safeguards for HIT implementation, given the vast cultural and environmental differences that can exist even within a country [80]. In addition, limitations in assembly and association may adversely influence public health initiatives and health policy [78]. These concepts can provide a more nuanced assessment of the data security risk.
2. Economic Freedom includes economic liberty, sound money, property rights, international trade, and regulation of financial institutions. These indices have been gathered from the Human Freedom Index 2019 by the Cato Institute as an index value between 0 and 1 [72]. Economic freedom provides insight into the financial opportunities for individuals and organizations. In societies with high economic freedom there may be additional economic incentives to develop robust HIT [81]. There may also be opportunities for private and public-private partnerships to enhance data security [82].
3. Global Freedom is a concept developed by Freedom House, a US-based nonprofit focused on promoting democracy, and published in their annual Freedom in the World Report since 1973 [79,83]. It is a quantitative and qualitative assessment of political rights and civil liberties in countries and territories around the world, represented as a weight score on a scale from –4 to 100. Evaluations of a country’s electoral process, political participation, government functioning, associational rights, rule of law, and personal autonomy make up the global freedom score. Freedom House also publishes other relevant indices such as the Internet Freedom Score and Democracy Score, but these cover a significantly smaller number of the world’s countries (65 countries and 29 countries, respectively) and thus may not be as helpful in creating a standard analytical approach.

### Partnership and Trust

An increasing number of partnerships have been developing between high-income countries and LMICs to address the global burden of disease. The success of these projects requires strong partnerships, which involves establishing rapport [84]. Relationship building and trust have been shown to be critical in navigating pragmatic obstacles as well as cultural and logistical boundaries [85,86]. Wagner et al [85] highlight how local coordinators and hosting communities are vital for the execution of international projects, and therefore establishing relationships and promoting ongoing collaboration are imperative to the success of global health efforts.

The strength of partnerships may be a moderating factor for concerns around patient data misuse. Organizations should objectively evaluate their global health partnerships. However, there is limited literature on global health partnership assessment tools. Instruments such as the Partnership Assessment Toolkit do exist, but studies need to be conducted to better understand their uses, limitations, and effectiveness [87,88]. To our knowledge, there are no tools that specifically address health data concerns. Given that each partnership is unique and influenced by a number of factors, we propose that this domain should be a self-assessment completed by the collaborating organizations. Relevant questions to explore include details about the in-country partner, the length of time the partnership has existed, the scope of the partnership, sensitive personal data collection, and relevant data security expertise resources available to the partners. Textbox 2 presents a list of potential questions to include in a self-assessment.

**Potential questions for Partnership and Trust self-assessment.**

**Questions to include in a self-assessment**

- How long have your organizations worked together?
- How long has your partner been active in-country?
- How much experience does your partner have with health data?
- Is your local partner in good standing in-country?
- What is your organization’s reputation in-country?
- Is an official government entity with oversight over health, health care, or data involved in your project? If not, should they be?
- Are there known examples of health data misuse in the country?
- In your partnership, who owns the data?
- Who is responsible for data security?
- What, if any, sensitive patient data are being collected or used?
- What physical, technical, and procedural measures have been taken to safeguard patient data?
- What data safety and monitoring measures will be put in place?
- Do you and your partner have the relevant experience to serve as data stewards?

### Conceptual Framework

**Figure 3:**
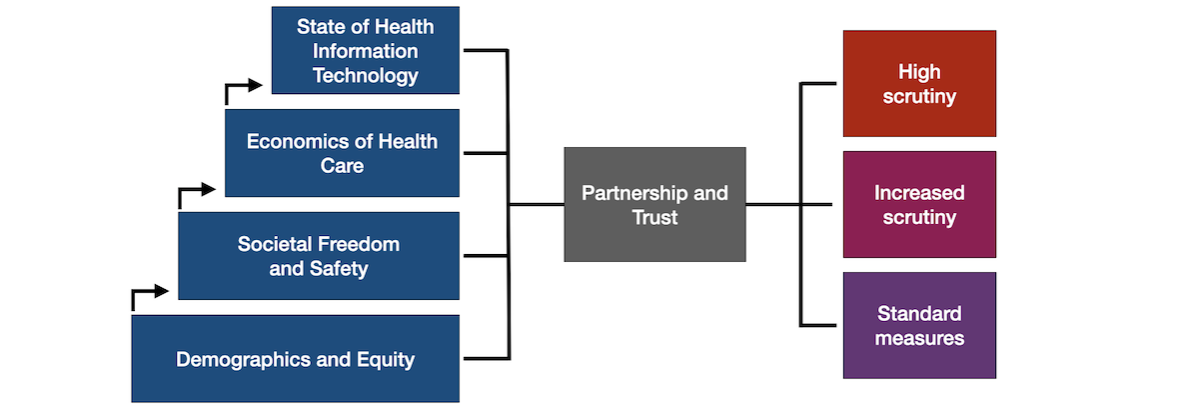
Four of the domains build on each other (Demographics and Equity, Economics of Health Care, Societal Freedom and Safety, and State of Health Information Technology). These are moderated by the fifth domain, Partnership and Trust. Together, these domains can be evaluated to produce a risk stratification for global health data partnerships and health information technology projects.

Figure 3 shows the relationship among these 5 domains and how they might be leveraged to provide a risk stratification of global health data partnerships. This type of conceptual framework has been leveraged to address various issues in informatics, such as the development of global health networks, patient safety, and conceptual models for research [89-91]. The value of these conceptual frameworks is in laying out all the components of a given issue, exploring their interrelationships, and identifying the emerging complexity [92]. In the current framework, *Demographics and Equity* and *Societal Freedom* provide a foundational understanding of a given country. *Economics of Health Care* can be understood within that context, and the *State of HIT* is influenced by, and builds upon, all 3 domains. *Partnership and Trust* is a moderating factor for all other variables. A long-standing, effective partnership with high levels of trust and cooperation may overcome a number of deficiencies in other domains, whereas an unstable or ineffective partnership may suffer from serious data concerns despite an otherwise favorable environment. The latter case is often the cause for HIT implementation failures in high-income countries [93].

## Discussion

### Overview

As HIT deployments continue to progress in LMICs, data security concerns will become more prevalent. The development of this conceptual framework is an attempt to better understand the many variables that might affect health data security in a given country. There are a number of existing models for assessing the maturity of HIT and data security [94]. However, most of these models have been developed or applied in high-income countries and make assumptions about the legal, regulatory, and technical capacity already in place; these assumptions often do not hold true in LMICs. The health systems of high-income countries (and the social, political, and economic forces that support them) can vary significantly from those in LMICs. For example, in the review by Tarhan et al [94], the authors provide the full list of countries in which the maturity models they reviewed were developed and applied. The average WHO health performance index of these countries was 0.813 (SD 0.17), whereas the average health performance index of CIS countries is 0.612 (SD 0.12; *P*=.001 by 2-tailed, 2 sample *t* test) [38]. Therefore, it may not be meaningful to apply existing maturity models to LMICs; to our knowledge, there are no models explicitly developed for low-resource settings.

The goal of our research is ultimately to develop an assessment and decision support tool that organizations can use in their global health partnerships. In the first version of this tool, we anticipate a more qualitative approach in which organizations use these domains to guide them in conducting a thorough evaluation of projects and partnerships. A second version of the tool will have a more quantitative component; 4 of the 5 key domains we identified use publicly available indices that could be integrated into a mathematical model that describes health data risk. Multimedia Appendix 5 presents the values for a sampling of indices across the domains of *State of HIT*, *Economics of Health Care*, *Demographics and Equity*, and *Societal Freedom*. We have included 9 CIS countries for which data are available (Armenia, Azerbaijan, Belarus, Kazakhstan, Kyrgyzstan, Moldova, Russia, Tajikistan, and Ukraine). In addition, data have been aggregated for several other countries to serve as reference points for the 4 domains of interest. These range from countries that have a track record of excellence in public health (Germany, France, Switzerland, South Korea, and Japan) to countries that have made significant advances in HIT (Estonia) and modernized their health care systems in other geographic regions (China and South Africa), as well as the United States. For the fifth domain, *Partnership and Trust*, additional research and validation will be required to finalize the self-assessment questionnaire.

The concept of bias was a recurring theme in many of the sources we evaluated. During the COVID-19 epidemic, the disproportionate impact that bias, discrimination, and racism can have on health outcomes was highlighted at scale [95]. These social biases can easily be translated into HIT; data sets, algorithms, and predictive models are all subject to both explicit and implicit bias [70]. The types of individuals who are included in data sets, the data elements that are and are not collected, the variables that are highlighted, and the outcomes that are selected for can all result in HIT applications that adversely affect care delivery and further drive health disparities among marginalized groups [70,96,97]. Although not directly linked to data security considerations (except to the extent to which security failures may adversely affect members of marginalized groups, as discussed previously in other domains), we believe that an awareness of bias is critical in any global health data partnership. Additional research is needed to identify approaches to measure and account for biases that may differ across settings.

### Limitations

Our study includes several limitations. Although we reviewed the literature to inform our research and approach, there is still a need for a comprehensive systematic literature review to be conducted and published. Given the evolving nature of the subject, a scoping review methodology would be appropriate; our research team is preparing to embark on this project [98]. Our proposed domains reflect our research and experience but need further validation from the broader community of health care, HIT, and public policy professionals. Our conceptual framework has not been tested qualitatively or quantitatively against real-world examples; thus, it remains to be seen if it can meaningfully capture the complexities and nuances of health data security. Finally, it is unclear if our proposed indices will result in a useful quantitative model of risk; further analysis is required.

### Next Steps

To advance our research agenda, we plan to engage in the following activities:

1. Validate our conceptual framework: Additional work is needed to validate our proposed framework. We will share our framework publicly to gather both formal and informal feedback from stakeholders around the world. We will also begin the work of applying the framework to real-world examples in collaboration with local experts to test its internal and external validity. Further literature review and qualitative research will be needed to finalize the Partnership and Trust self-assessment.
2. Develop a qualitative assessment tool: Once we have finalized and validated the framework, we will develop a qualitative assessment tool that other organizations can use to evaluate their existing data partnerships. This stage will not only provide additional external validation and refinement of the framework, but will also provide the opportunity to conduct user-centered design research to improve the usability of the tool and related documentation.
3. Develop a quantitative model: We plan to work with our data science colleagues to use both traditional statistical methods and more modern machine learning approaches to develop a quantitative model of our conceptual framework. This will require extensive validation, but if successful, it may result in a risk stratification that could conceivably be calculated for every country, needing only the Partnership and Trust self-assessment to provide local context.
4. Dissemination of findings: Our overall goal is to support how organizations make decisions around global health data partnerships. The current framework, assessment tools, and quantitative models are all intended to help organizations make the best decisions possible in terms of safeguarding patient data in LMICs. To that end, we intend to use multiple avenues to disseminate our work, including publications, presentations, webinars, and white papers. We plan to collaborate with universities and nongovernmental organizations to help them to implement the conceptual framework and associated tools.

### Conclusions

Global health HIT partnerships have the potential to have a positive impact in LMICs, leveraging the resources and knowledge of partner organizations to build in-country capacity and expertise. However, there are gaps in the legal, technical, and regulatory environments in many LMICs, increasing the risk of possible health data misuse, particularly among marginalized and vulnerable populations. Our conceptual framework helps to identify key domains that may have an impact on health data security considerations in global health partnerships. Additional research is needed to further validate and improve the framework. We encourage global health, HIT, and health care professionals to participate in improving this framework. In the future, we hope to be able to leverage our framework to create assessment and decision-making tools that can be used to evaluate risk in other global health initiatives such as clinical and academic partnerships, pandemic control, and emergency response operations.

## Appendix

Multimedia Appendix 1 State of health information technology domain.

Multimedia Appendix 2 Economics of healthcare domain.

Multimedia Appendix 3 Demographics and equity domain.

Multimedia Appendix 4 Societal freedom and safety domain.

Multimedia Appendix 5 Example index values for CIS and reference countries.

## Abbreviations

CIS: Commonwealth of Independent States
EHR: electronic health record
GBD: Global Burden of Disease
GDP: gross domestic product
HIT: health information technology
LMIC: low- and middle-income country
WHO: World Health Organization
