# Supplementary material for: Assessing Health Data Security Risks in Global Health Partnerships: Development of a Conceptual Framework
Source: JMIR Form Res. 2021 Dec 8;5(12):e25833. doi: 10.2196/25833 (PMC8701669; doi:10.2196/25833)
Supplement: Multimedia Appendix 1 [file formative_v5i12e25833_app1.pdf]

Variable Name	Subdomain	Description	Value	Source
National EHR system	Electronic Health Records	If a country has a national EHR system.	Yes, No, N/A	World Health Organization [6]
Legislation governing the use of the national EHR system	Electronic Health Records	If the country has legislation regulating the use nations EHRs.	Yes, No, N/A	World Health Organization [6]
Implementation of EHR systems by primary, secondary, or tertiary care facilities	Electronic Health Records	If primary (clinics and health centers), secondary (hospitals, emergency care), or tertiary care (specialized care) facilities use EHRs.	Yes, No, N/A	World Health Organization [6]
National eHealth policy or strategy	eHealth Foundations	If the country has a national strategy or policy for electronic health info.	Yes, No, N/A	World Health Organization [6]
National health information system (HIS) policy or strategy	eHealth Foundations	If the country has a national strategy or policy for health information systems.	Yes, No, N/A	World Health Organization [6]
National telehealth policy or strategy	eHealth Foundations	If the country has a national strategy or policy for telehealth.	Yes, No, N/A	World Health Organization [6]
Public funding for eHealth	eHealth Foundations	If there is public funding for eHealth.	Yes, No, N/A	World Health Organization [6]
Private or commercial funding for eHealth	eHealth Foundations	If there is private of commercial funding for eHealth.	Yes, No, N/A	World Health Organization [6]
Donor/non-public funding for eHealth	eHealth Foundations	If there is donor funding for eHealth.	Yes, No, N/A	World Health Organization [6]
Public-private partnerships for eHealth	eHealth Foundations	If public-private partnerships exists for developing eHealth.	Yes, No, N/A	World Health Organization [6]
Defined medical jurisdiction, liability or reimbursement of eHealth services such as telehealth	Legal Frameworks for eHealth	If there are medico-legal guidelines and definitions regarding reimbursement, laibility, and jurisdiction for eHealth services.	Yes, No, N/A	World Health Organization [6]
Policy or legistlation addressing patient safety and quality of care based on data quality, data transmission standards or clinical competency criteria	Legal Frameworks for eHealth	If patient safety is addressed based on data and clinical standards.	Yes, No, N/A	World Health Organization [6]
Policy or legistlation protecting the privacy of personally identifiable data of individuals irrespective of whether it is in paper or digital format	Legal Frameworks for eHealth	If hardcopy and digital data is protected and privacy is accounted for.	Yes, No, N/A	World Health Organization [6]
Policy or legistlation protecting the privacy of individuals' health-related data held in electronic format in an EHR	Legal Frameworks for eHealth	If EHR data is protected and privacy is accounted for.	Yes, No, N/A	World Health Organization [6]
Policy or legistlation governing the sharing of digital data between health professionals in other health services in the same country through the use of an EHR	Legal Frameworks for eHealth	If there is governance of sharing electronic data in the same country.	Yes, No, N/A	World Health Organization [6]
Policy or legistlation governing the sharing of digital data between health professionals in health services in other countries through the use of an EHR	Legal Frameworks for eHealth	If there is governance of sharing electronic data within different countries.	Yes, No, N/A	World Health Organization [6]
Policy or legistlation governing the sharing of personal and health data between research entities	Legal Frameworks for eHealth	If there is governance of sharing electronic data between resarch entities.	Yes, No, N/A	World Health Organization [6]
Policy or legislation allowing individuals electronic access to their own health-related data when held in an EHR	Legal Frameworks for eHealth	If patients are allowed electronic access to their own data.	Yes, No, N/A	World Health Organization [6]
Policy or legislation allowing individuals to demand their own health-related data be corrected when held in an EHR if it is known to be inaccurate	Legal Frameworks for eHealth	If patients are able to correct their health data.	Yes, No, N/A	World Health Organization [6]
Policy or legislation allowing individuals to demand the deletion of health-related data from their EHR	Legal Frameworks for eHealth	If patients are able to delete their health data.	Yes, No, N/A	World Health Organization [6]
Policy or legislation allowing individuals to specify which health-related data from their EHR can be shared with health professionals of their choice	Legal Frameworks for eHealth	If patients can govern the sharing of their data with health professionals.	Yes, No, N/A	World Health Organization [6]
