# Supplementary material for: Assessing Health Data Security Risks in Global Health Partnerships: Development of a Conceptual Framework
Source: JMIR Form Res. 2021 Dec 8;5(12):e25833. doi: 10.2196/25833 (PMC8701669; doi:10.2196/25833)
Supplement: Multimedia Appendix 2 [file formative_v5i12e25833_app2.pdf]

Variable Name	Subdomain	Description	Value Type	Source
Population	Population	The country's population size.	Number	United Nations [26]
GDP	Healthcare Expenditure	The gross domestic product of the country.	Number (Millions)	World Bank [27]
GDP Per Capita	Healthcare Expenditure	The gross domestic product of the country based on the population size.	Number	World Bank [28]
Economic Growth	Healthcare Expenditure	Economic growth: the rate of change of real GDP, 2018	Number (-8 to 10)	World Bank [29]
Healthcare spending as a percent of GDP	Healthcare Expenditure	The gross domestic product expenditure percentage on healthcare.	Number	World Bank [30]
Health Expenditures per capita	Healthcare Expenditure	The healthcare expenditure of the gross domestic product based on the population size.	Number (0-15000)	World Bank [31]
Doctors per capita	Healthcare Structure	The amount of doctors based on the population.	Number: Doctors per 1,000 people	World Bank [32]
Hospital beds	Healthcare Structure	The amount of hospital beds in a country.	Number: Beds per 1,000 people	World Bank [33]
Healthcare Access	Healthcare Structure	An evaluation of the country's quality and access to healthcare.	Number (0-100)	Global Burden of Diseases HAQ Index [34]
Public healthcare system	Healthcare Structure	If the country has a public or private healthcare system.	Text (categorical)	The International Social Security Association [35]
Universal Health Coverage	Healthcare Cost	How close the country is to universal health coverage, expressed as a number where 100 is complete universal coverage.	Number (0-100)	World Bank [36]
Share of Out-of-Pocket healthcare expenditure	Healthcare Cost	Out-of-pocket expenditure on healthcare as percent of total healthcare expenditure.	Number (percentage 0-100)	World Bank [37]
WHO Health System Ranking (Health Performance Index, 2000)	Healthcare Quality	The ranking of the country based on the development and success of the health system.	Ranking, 1-191	World Health Organization [38]
Infant mortality rate	Healthcare Quality	The number of infant deaths per 1,000 live births.	Number (rate)	Central Intelligence Agency [39]
Maternal mortality rate	Healthcare Quality	The number of pregnancy related deaths per 100,000 live births.	Number (rate)	World Health Organization [40]
Life Expectancy	Healthcare Quality	The average number of years a newborn would live if age-specific mortality rates in the current year were to stay the same throughout its life.	Number	World Health Organization [41]
DPT Immunization rate	Healthcare Quality	Percent of children ages 12-23 months with DPT immunization	Number (percent)	World Health Organization [42]
Diarrheal disease death rate	Healthcare Quality	The annual number of deaths from diarrheal diseases per 100,000 people.	Number (rate)	Global Burden of Diseases Results Tool [43]
