# Supplementary material for: Assessing Health Data Security Risks in Global Health Partnerships: Development of a Conceptual Framework
Source: JMIR Form Res. 2021 Dec 8;5(12):e25833. doi: 10.2196/25833 (PMC8701669; doi:10.2196/25833)
Supplement: Multimedia Appendix 3 [file formative_v5i12e25833_app3.pdf]

Variable Name	Subdomain	Description	Value	Source
Population age structure	Population	The structure of the population demographics.	Number (percent)	World Bank [49][50][51]
Population Density (Urban)	Population	The density of the country's urban population.	Number (thousands)	United Nations [52]
Population Density (Rural)	Population	The density of the country's rural population.	Number (thousands)	United Nations [52]
Wealth Inequality	Social Structure	A quantification of social inequality in a nation.	Number (percent)	World Bank [53]
Percent of population living in Poverty	Social Structure	The percent of the country's population living below the poverty line.	Number (percent)	World Bank [54]
Measure of Decentralization	Social Structure	A measure of how centralized the government is fiscally, administratively, and politically.	Number (0-1)	World Bank [55]
Public Trust	Social Structure	A quantification of the trustworthiness of the government, media, NGOs, and business in a nation based on the Edelman Trust Barometer.	Number (1-100)	Edelman Global Communications Firm [56]
Human Development Index (HDI)	Community Development	A measure of human development based on: health and longevity, knowledge, and standard of living.	Number (0-1)	United Nations [57]
Internet subscribers	Community Development	Fixed broadband internet subscribers per 100 people, 2018	Number (0-100)	World Bank [58]
Access to Electricity	Community Development	Access to electricity, percent of the population, 2017	Number (percent)	World Bank [59]
Literacy rate (ages 15 and older)	Community Development	Share of population, 15 years and older, with education.	Number (percent)	World Bank [60]
Social Media Penetration	Community Development	A measure of social media penetration.	Number (percent)	StatCounter [61]
