# Supplementary material for: Assessing Health Data Security Risks in Global Health Partnerships: Development of a Conceptual Framework
Source: JMIR Form Res. 2021 Dec 8;5(12):e25833. doi: 10.2196/25833 (PMC8701669; doi:10.2196/25833)
Supplement: Multimedia Appendix 4 [file formative_v5i12e25833_app4.pdf]

Variable Name	Subdomain	Ranking	Description	Source
Overall Human Freedom	Human Freedom Index	1-10	A general value combining all the indices.	Cato Institute [72]
Overall Freedom Rank	Human Freedom Index	1-162	A general value combining all the indices with respect to the global context.	Cato Institute [72]
Overall Economic Freedom	Economic Freedon Index	1-10	A general value of an individual's ability to acquire property without force, fraud or theft and have the ability to exchange, use, or give property.	Cato Institute [72]
Overall Personal Freedom	Personal Freedom Index	1-10	A general value of a person's individual freedom.	Cato Institute [72]
Rule of Law	Personal Freedom Index	1-10	A broad concept which includes: due process, government accountability, and justice (procedural, civil, and criminal).	Cato Institute [72]
Security and Safety	Personal Freedom Index	1-10	A measure of crimes commited including: physical assault, kidnapping, killing, or the violation of an inidividual's physical integrity or safety.	Cato Institute [72]
Movement	Personal Freedom Index	1-10	The ability to move domestrically and internationally, and women's ability to move outside of the house.	Cato Institute [72]
Religious Freedom	Personal Freedom Index	1-10	The right to establish and operate a religious organization, harassment and physical hostility towards religious freedom, and legal restrictions.	Cato Institute [72]
Association, Assembly, and Civil Society	Personal Freedom Index	1-10	An individual’s right to join and form an organization--political, professional, educational, sporting, or cultural.	Cato Institute [72]
Expression and Information	Personal Freedom Index	1-10	A broad category which includes personal freedom, the press, and the internet.	Cato Institute [72]
Identity and Relationships	Personal Freedom Index	1-10	The ability to establish gender identity and intimate relationships.	Cato Institute [72]
Global Freedom Score	Global Freedom Index	-4-100	A rating of political rights and civil liberties.	Freedom House [79]
