# Supplementary material for: Assessing Health Data Security Risks in Global Health Partnerships: Development of a Conceptual Framework
Source: JMIR Form Res. 2021 Dec 8;5(12):e25833. doi: 10.2196/25833 (PMC8701669; doi:10.2196/25833)
Supplement: Multimedia Appendix 5 [file formative_v5i12e25833_app5.pdf]

Domains	Demographics and Equity			Societal Freedom			Economics of Healthcare					State of HIT				
CIS Countries (2019)	Percent of population living in poverty	Human Development Index (HDI)	Literacy Rate	Personal Freedom	Economic Freedom	Global Freedom	Per Capita GDP	Healthcare Spending as Percent GDP	Health Performance Index (HPI)	Infant Deaths per 1,000 live births	Pregnancy related deaths per 100,000 live births	National EHR System	National eHealth policy or strategy	Public-private partnerships for eHealth	Addresses patient safety and quality of care	Ownership and correction of health-data by individuals
Armenia	23.5	0.760	100	7.15	7.7	53	$4,545.38	10.0	0.630	12.7	25	No	Yes	No	No	N/A
Azerbaijan	6.0	0.754	100	6.1	6.34	10	$4,694.70	6.7	0.626	23.8	25	Yes	Yes	Yes	No	N/A
Belarus	5.6	0.817	100	6.65	6.64	19	$6,619.69	5.9	0.723	3.6	4	Yes	No	No	No	No
Kazakhstan	2.5	0.817	100	6.69	7.1	23	$9,181.29	3.4	0.752	19.6	12	Yes	Yes	Yes	Yes	N/A
Kyrgyzstan	22.4	0.674	100	7.05	6.92	38	$1,287.59	6.4	0.455	25.9	76	No	No	Yes	No	No
Moldova	23.0	0.711	99	7.2	6.66	60	$2,890.73	7.5	0.639	11.9	23	Yes	Yes	No	No	Yes
Russia	12.9	0.824	100	5.9	6.78	20	$11,228.26	5.3	0.544	6.8	25	Yes	Yes	Yes	Yes	No
Tajikistan	26.3	0.656	100	5.49	6.05	9	$874.58	7.0	0.428	31.8	32	Yes	N/A	No	Yes	No
Ukraine	1.3	0.750	100	6.56	5.96	62	$3,659.00	6.8	0.708	7.8	24	No	Yes	Yes	No	No
Representative Countries																
China	0.6	0.758	97	5.92	6.42	10	$10,650.10	5.0	0.485	12	27	Yes	Yes	No	No	No
Estonia	21.7	0.608	100	9.02	7.89	94	$23,413.46	6.5	0.714	3.8	9	Yes	Yes	No	Yes	Yes
France	13.4	0.891	-	8.69	7.35	90	$42,555.41	11.5	0.994	3.1	8	N/A	N/A	N/A	N/A	N/A
Germany	16.0	0.939	-	9.25	7.82	94	$47,681.70	11.1	0.902	3.4	6	N/A	N/A	N/A	N/A	N/A
Guatemala	59.3	0.651	81	6.56	7.57	52.00	$4,620.00	6.0	0.713	21.3	88	No	No	No	No	No
Japan	-	0.915	-	8.7	7.86	96	$42,669.41	10.8	0.957	2	5	No	Yes	Yes	Yes	N/A
South Africa	55.5	0.705	87	7.55	6.61	79	$6,127.90	8.1	0.319	31	138	No	Yes	Yes	Yes	N/A
South Korea	-	0.906	100	8.81	7.59	83	$31,752.92	7.3	0.759	3	11	N/A	N/A	N/A	N/A	N/A
Switzerland	14.6	0.946	-	9.24	8.4	96	$83,265.00	12.2	0.916	3.6	5	No	Yes	Yes	Yes	No
US	-	0.920	-	8.72	8.19	86	$67,833.92	17.2	0.838	5.8	14	No	Yes	No	Yes	Yes
